# Adiponectin and Cognitive Decline

**DOI:** 10.3390/ijms21062010

**Published:** 2020-03-16

**Authors:** Maria Rosaria Rizzo, Renata Fasano, Giuseppe Paolisso

**Affiliations:** Department of Advanced Medical and Surgical Sciences, University of Campania “Luigi Vanvitelli”, Piazza Miraglia 2, 80138 Naples, Italy; renatafasano2@gmail.com (R.F.); giuseppe.paolisso@unicampania.it (G.P.)

**Keywords:** adiponectin, cognitive function, MCI, dementia

## Abstract

Adiponectin (ADPN) is a plasma protein secreted by adipose tissue showing pleiotropic effects with anti-diabetic, anti-atherogenic, and anti-inflammatory properties. Initially, it was thought that the main role was only the metabolism control. Later, ADPN receptors were also found in the central nervous system (CNS). In fact, the receptors AdipoR1 and AdipoR2 are expressed in various areas of the brain, including the hypothalamus, hippocampus, and cortex. While AdipoR1 regulates insulin sensitivity through the activation of the AMP-activated protein kinase (AMPK) pathway, AdipoR2 stimulates the neural plasticity through the activation of the peroxisome proliferator-activated receptor alpha (PPARα) pathway that inhibits inflammation and oxidative stress. Overall, based on its central and peripheral actions, ADPN appears to have neuroprotective effects by reducing inflammatory markers, such as C-reactive protein (PCR), interleukin 6 (IL6), and Tumor Necrosis Factor a (TNFa). Conversely, high levels of inflammatory cascade factors appear to inhibit the production of ADPN, suggesting bidirectional modulation. In addition, ADPN appears to have insulin-sensitizing action. It is known that a reduction in insulin signaling is associated with cognitive impairment. Based on this, it is of great interest to investigate the mechanism of restoration of the insulin signal in the brain as an action of ADPN, because it is useful for testing a possible pharmacological treatment for the improvement of cognitive decline. Anyway, if ADPN regulates neuronal functioning and cognitive performances by the glycemic metabolic system remains poorly explored. Moreover, although the mechanism is still unclear, women compared to men have a doubled risk of developing cognitive decline. Several studies have also supported that during the menopausal transition, the estrogen reduction can adversely affect the brain, in particular, verbal memory and verbal fluency. During the postmenopausal period, in obese and insulin-resistant individuals, ADPN serum levels are significantly reduced. Our recent study has evaluated the relationship between plasma ADPN levels and cognitive performances in menopausal women. Thus, the aim of this review is to summarize both the mechanisms and the effects of ADPN in the central nervous system and the relationship between plasma ADPN levels and cognitive performances, also in menopausal women.

## 1. Introduction

Adiponectin (ADPN) is a plasma protein that belongs to the complement 1q family and it is secreted by adipose tissue [1,2]. ADPN has pleiotropic effects, such as anti-diabetic effects, increased insulin sensitivity of target organs [3], anti-inflammatory, and anti-atherogenic properties [4,5]. For these characteristics, ADPN is a protective factor in conditions such as obesity, type 2 diabetes, and cardiovascular diseases [6]. ADPN circulates in the blood in three forms: high, medium, and low molecular weight ADPN (HMW-, MMW-, and LMW-Ad) (6). There are three ADPN Receptors (AdipoR1, AdipoR2, and T-cadherin) prevalently expressed in the liver, muscles, heart, adipose tissue, pancreas, and brain; these receptors show a different affinity for specific ADPN forms [7,8,9]. In particular, ADPN activates 5′–AMP-activated protein kinase (AMPK), which functions as a sensor of intracellular energy state and phosphorylates acetyl-CoA carboxylase (ACC), causing the augmentation of fatty acid oxidation and glucose uptake in the liver, muscle cells, and adipocytes [7,8,9].

In addition to improving both insulin sensitivity and glucose metabolism, ADPN shows anti-inflammatory activity, which is achieved by counteracting inflammatory cytokines and suppressing Tumor Necrosis Factor α (TNFα) production through the inhibition of p38 mitogen-activated protein kinase (p38MAPK) and TNFα-mediated inflammatory signaling from macrophages [10]. Therefore, ADPN provides protection from oxidative stress-mediated cytotoxicity by reducing the production of reactive oxidative stress (ROS) through AMPK signaling.

Indeed, oxidative stress and ROS have a deleterious effect on various cellular processes and on various organs of the human body, activating multiple cellular signaling pathways, such as AMPK, which affects cellular processes because it causes different cellular outcomes, such as apoptosis, proliferation, and autophagy. Therefore, drugs used as modulators of AMPK activity could represent a clinical opportunity for the treatment of diseases, as a metabolic dysfunction, cancer, and neurodegeneration [11]. During recent years, different epidemiological and clinical studies showed the important role of oxidative stress on the human organism. In fact, there is an increase in many diseases related to oxidative stress, such as oral diseases (periodontitis), as well as other chronic diseases, such as cardiovascular, pancreatic, gastric, liver and neurodegenerative diseases and / or cancer, chronic inflammation, stroke and aging [11]. Although ADPN action in the periphery is more known, very little is known about the presence and effects of ADPN in the brain. Initially, the opinion was that ADPN was initially thought not to be present in the brain, and not detectable in CSF [12,13], probably because of its inability to cross the blood-brain barrier (BBB). Subsequently, some studies demonstrated that an i.v. injection of ADPN in mice reduced glucose and lipid levels, and body weight as other studies demonstrated ADPN stimulated AMPK in the hypothalamus of mice producing an increase in food intake and a decrease energy expenditure, thus suggesting that ADPN acts centrally [14].

Therefore, in addition to internal factors such as insulin resistance and oxidative stress, external factors such as lifestyle could also influence the production and action of ADPN. In fact, diet, physical activity, but also the natural dietary supplements exert neuroprotective effects [15].

## 2. Adiponectin in the Brain

ADPN is not expressed in the brain but it enters the brain through peripheral circulation, crossing the blood–brain barrier (BBB), modulating and signaling its action through its receptors [1]. In fact, ADPN, partly through AdipoR1, carries out neuroprotective action against the damage caused by unsuitable lifestyles, such as a high-fat diet. AdipoR1 and AdipoR2 are highly expressed in the brain and, in particular, in various areas, including the hypothalamus, brain stem, hippocampus, and cortex. In the hypothalamus through AdipoR1, ADPN regulates food consumption and energy expenditure, while in the hippocampus it seems to promote neurogenesis through AdipoR1 and synaptic function through AdipoR2 [1]. Conversely, the interactions between ADPN and T-cadherin in the brain is unclear. T-cadherin is a receptor expressed in the brain (1), but mainly in the cardiovascular system, with a cardioprotective role, that regulates cell growth, proliferation, and migration [1].

Although it seemed that ADPN did not cross the BBB [10], in recent years, high-sensitive ELISA systems have allowed us to analyze ADPN in CSF [16]. Several reports showed that ADPN was detectable in the CSF of patients with unspecified neurological disorders, including stroke depression, Alzheimer’s disease, and multiple sclerosis, with evidence that only low molecular weight ADPN was detectable in CSF [17]. In fact, only this form can cross the BBB and have a role in the central nervous system (CNS) [1]. Anyway, the concentration of ADPN in the CSF is approximately 1000-fold lower than the blood concentration [18]. Besides, contradictory results have been published. However, taken together, data from the studies show that ADPN in human CSF is far below the level expected by the permeability of the BBB, indicating that ADPN enters the brain much less efficiently than other proteins, thus supporting recent data that exclude ADPN transport to the CSF [18].

Furthermore, recent preclinical studies investigated ADPN involvement in different physiological functions. In fact, the ADPN receptor signal in the brain appears to play a role not only in energy homeostasis but also in neuronal excitability and synaptic plasticity, in order to reduce both amyloid-β (Aβ) and promote neuroprotection and the best regulation of glial cell activation [16,17].

Indeed, Chan et al. demonstrated that ADPN is protective against oxidative stress-induced cytotoxicity in human neuroblastoma cells affected by Amyloid-β (Aβ) neurotoxicity [19]. Aβ derives from the cleavage of amyloid precursor protein (APP) by two secretases. There are different Aβ forms. Aβ oligomers forms are directly toxic to neurons, impair neuronal synaptic transmission, and induce uncontrolled ion flux. Aβ causes oxidative stress, inflammation, and mitochondrial damage resulting in neuronal degeneration and death. The ADPN neuroprotective action against Aβ neurotoxicity is done through the AMPK activation and suppression of NF-kB activation [20,21]. Consequently, this effect appears related to the reduction of the inflammation cascade by several mechanisms [1]. In fact, ADPN reduces inflammatory markers, such as C-reactive protein (PCR), interleukin 6 (IL6), TNFα, and increases the expression of anti-inflammatory molecules, such as interleukin-10 (IL10), in vivo and in vitro [1,20,21].

Studies in vitro showed that ADPN increases proliferation in hippocampal progenitor cells, by activating the p38MAPK, while an ADPN-deficit induces neurogenesis reduction in adults (18). In particular, ADPN might directly regulate the activation state of microglia [22]. In fact, astrocytes and macrophages also possess ADPN receptors, suggesting that ADPN can regulate cerebral and peripheral inflammation. Waragai et al. confirmed this possibility by supporting the view that ADPN might to have a critical role in the balance between M1 and M2 microglial polarization states [23].

As we know, AMPK displays also a strong relationship with p38MAPK apoptosis [24]. Many pieces of evidence show that MAPK is also involved in hyperglycemia-induced apoptosis [25]. However, the precise mechanisms remain poorly understood. Wang et al. investigated the role of ADPN on p38 MAPK and AMP-activated protein kinase (AMPK) in high glucose conditions in rat tubular NRK-52E cells [26]. They demonstrated that ADPN exerts a crucial protective role against apoptosis induced by high glucose via the AMPK/p38MAPK pathway [26].

However, ADPN was found to modulate glucose metabolism in hippocampal neurons, increasing glucose uptake, glycolysis, and ATP production rates [27]. It is suggestive to hypothesize that the protective effect of ADPN at the neurological level is linked to its insulin-sensitizing action [28].

Although ADPN deficiency is associated with peripheral insulin resistance in mice and humans, causing diabetes [28,29], whether ADPN is associated with cerebral insulin sensitivity has not been documented. In the meantime, T2DM patients with lower serum ADPN levels have a lower mean hippocampal volume than T2DM patients with normal ADPN levels [30].

In a recent study, the authors investigated if chronic ADPN deficiency was associated with cerebral insulin resistance in knockout (ADPN-KO) mice. They demonstrated that ADPN deficiency was associated with cerebral insulin resistance and deregulated insulin signaling. Moreover, the ADPN-KO mice also exhibited memory decline associated with increased Aβ production, Tau phosphorylation, neuroinflammation, and neurodegeneration, representing the typical dementia alterations [31].

In addition, a reduction in insulin signaling is known to be associated with cognitive impairment. In fact, insulin is needed for learning, memory, and synaptic plasticity mainly in the brain of older patients who are more vulnerable to lower glucose levels [1,28].

## 3. Cognitive Function and Dementia

There is ample evidence that cognitive functions change simultaneously across the lifespan due to the aging of brain structure. The cognitive functions are complex. Therefore, all cognitive functions are divided into multiple specific cognitive domains (attention, memory, language, visuospatial abilities, and executive functioning/reasoning) [32]. Consequently, age-related changes in cognition are not uniform across all cognitive domains. Moreover, the degree of severity is not the same between all older individuals. Although the overall picture often seems to be of cognitive decline, enormous variability exists across individuals. In fact, there is significant heterogeneity among cognitive performances, going from normal cognitive aging to Mild Cognitive Impairment (MCI), Alzheimer Disease (AD) and Vascular Dementia (VD) [33]. MCI represents a stage of mild cognitive impairment that can increase the risk of later developing real dementia [34,35]. It can involve memory, language, thought, and judgment problems, which are greater than normal age-related changes, but are not serious enough to interfere with normal daily life activities. However, often, MCI represents a transition between the cognitive decline linked to the normal aging process and severe dementia, as AD [36]. AD is a neurodegenerative disorder, in which there is progressive astrogliosis, neuronal atrophy, and neuronal loss, in the hippocampus and cortex of the brain. This damage is caused both by the accumulation and deposition of oligomeric or fibrillar Aβ, and by the intracellular accumulation of neurofibrillary tangles containing hyperphosphorylated Tau protein and by the activation of the inflammation cascade [37,38].

Many studies described associations between ADPN and all-cause dementia, MCI, AD, VD and their clinical progression.

### 3.1. Adiponectin and Mild Cognitive Impairment (MCI)

Few studies have evaluated the circulating levels of ADPN in MCI and neurocognitive disorders. However, the association between ADPN levels and MCI has contrasting evidence [39]. In a small clinical sample, ADPN levels not differ between AD, VD, and patients with and without MCI. [40]. Teixeira AL [39] et al. evaluated ADPN serum levels in patients with MCI and dementia compared to cognitively healthy elderly individuals. The authors concluded that decreased ADPN levels were associated with cognitive dysfunction. Waragai et al. suggested that increased serum ADPN levels correlate with MCI, as a compensatory effect against neurodegeneration [23]. In contrast to previous findings, another report showed no significant difference in ADPN levels between a non-demented group and patients with dementia [40]. Nevertheless, the mechanism explaining the relationship between ADPN and impaired cognitive function is often related to the inflammatory cascade. More authors theorized that serum ADPN levels could indirectly influence cognitive performances through the modulation of several interrelated systemic factors and in particular the inflammation cascade [41]. Indeed, Jian et al. demonstrated that ADPN suppressed inflammatory response of microglia to amyloid-β oligomer (AβO) and ADPN deficiency may aggravate microglia-mediated neuro-inflammation in AD mice [41].

### 3.2. Adiponectin and Alzheimer Disease (AD)

The involvement of ADPN in AD is poorly understood, with divergent results showing a decrease [39,42], increase [23,24,25,26,27,28,29,30,31,32,33,34,35,36,37,38,39,40,41,42,43], or no significant changes [40,41,42,43,44]. However, in the past few years, several studies show the ADPN neuroprotective properties [1].

The same presence of AdipoR1 in the hypothalamus and in the Meynert basal nucleus suggests that ADPN may be involved in the control of the energy homeostasis pathway and to higher brain function [1]. Ng et al. have found impaired spatial memory and learning in mice ADPN-ko [45]. These mice also developed AD, displaying increased β-amyloid-42 fragments (Aβ42) levels in the hippocampus and frontal cortex, due to ADPN deficiency [28]. Bednarska-Makaruk [46] found that in the all-cause dementia group there are higher levels both of IL-6 and ADPN in AD and Mixed Dementia (MD).

In general, about the mechanisms involved in the pathogenesis of AD, several studies demonstrated that AMPK was deregulated in the brain of AD patients. AMPK could phosphorylate Tau protein causing changes in synaptic plasticity and memory. As a consequence, the disequilibrium between tau phosphorylation and phosphatase activities play a central role in AD development [47]. ADPN, through AMPK, can increase neuronal insulin sensitivity, increasing pAkt through AdipoR1. Conversely, the chronic deficit of ADPN inactivates AMPK, reducing insulin sensitivity and inducing AD in elderly mice with the development of cognitive deficits and psychiatric symptoms [1,48].

In addition, the same mechanisms linked to diabetes pathogenesis, such as oxidative stress, inflammation, mitochondrial failure, and insulin resistance, play an important role in the pathogenesis of AD [45]. Insulin resistance activates glycogen synthase3 (GSK3), which increases Aβ production and tau phosphorylation. Furthermore, due to enzymatic competition mechanism, hyperinsulinemia reduces the clearance of Aβ in the brain [45,49]. The aberrant activation of GSK3 was revealed by the reduction in pGSK3 S9 and by the increase in pGSK3 Y279 levels in elderly ADPN-KO mice. These findings explained the dramatic increase in phosphorylated Tau and in Aβ production. The increased neuronal apoptosis and reduced synaptic protein levels also indicated that chronic ADPN reduction was associated with neurodegeneration in aging [45,49].

Khemka et al. [43] have shown, using FDG-Pet, that brain glucose reduction in the early stage of AD, as well as weight loss and anorexia related to the decrease in leptin, increase the ADPN and the insulin levels in the blood. The authors also found a positive correlation between AD severity and serum insulin and ADPN levels. Two other studies described low ADPN levels in diabetic patients who had reduced gray matter volume, hippocampal volume, and glucose metabolism [23,50].

The increase in serum ADPN in AD may reflect systemic and compensatory mechanism against neurodegeneration [51]. However, this compensatory mechanism is limited. Some authors speculated that the sequestration of ADPN by pathological tau into NFTs may result in suppression of neurotoxicity of pathological tau.

So, currently, it is not yet known whether the changes in plasma ADPN levels in AD could be either the cause or consequence of AD onset.

### 3.3. Adiponectin and Vascular Dementia (VaD)

Vascular dementia is a neurocognitive disorder as a usual consequence of cerebrovascular disease and vascular risk factors. Epidemiological studies show a positive correlation between cardiovascular risk factors and cognitive decline [33,52], though there is a significant overlap between VaD and AD [33,34,35,36,37,38,39,40,41,42,43,44,45,52].

However, recent data show that a high level of ADPN reduces VaD risk, improves cerebrovascular dysfunction and cognitive decline, and decreases the risk of neuroinflammation [41]. It is likely that ADPN blocks the interaction between the endothelial cells and leukocytes in ischemia-reperfusion and also inhibits the secondary inflammation in cerebral ischemia-reperfusion [53].

Moreover, ADPN can have protective effects due to the stimulation of nitric oxide (NO) through AdipoR1 signaling and/or anti-inflammatory effects through the NF-kB pathway [35]. Against low ADPN serum levels are related to ischemic cerebrovascular disease and enhanced mortality post-stroke.

Considering these findings, the ADPN role may be due to anti-atherogenic proprieties and vasodilator effect in the vascular system, through decreasing the expression of atherogenic molecules and plaque formation in blood vessels, improving the vascularization and reducing infarct size in the ischemic brain [54].

## 4. Adiponectin and Risk Factors for Dementia

### 4.1. Obesity and Diabetes

Body composition changes with aging. Generally, the visceral adipose tissue increases during middle age with a trend to further increase compared to lean mass which appears reduced, thus configuring a state of sarcopenic obesity [55]. However, obesity is an independent risk factor for the development of cognitive decline, AD and its progression, morbidity, and mortality [56]. Some studies suggest that diet, lifestyle, and natural dietary supplements exert neuroprotective effects, thereby improving cognitive functions, by reducing oxidative stress and inflammation and improving brain insulin resistance [18]. On the contrary, an abnormal production and secretion of adipokines result from abnormal fat accumulation the and dysfunction of adipose tissue. Increased levels of pro-inflammatory adipokines, such as interleukin (IL)-1β, IL-6, TNFα, and leptin, and decreased levels of anti-inflammatory adipokines, such as ADPN, were found in obesity [2]. The resulting chronic inflammation state promotes the development of cardiovascular diseases, insulin resistance, and diabetes [57]. In obesity and diabetes, levels of circulating ADPN are decreased, contributing to insulin resistance worsening [58], which is also confirmed by elevated levels of phosphorylated IRS-1 at Ser616 and Ser636 [59] associated with a reduction in insulin receptor (IR) expression [60]. Therefore, in light of the fact that ADPN improves insulin sensitivity at the peripheral level and in the brain, it is of great interest to experiment with the use of ADPN as a treatment for AD. Interestingly, there is evidence from animal studies of AD and in vivo that already investigated the use of insulin-sensitizing agents [61,62,63], and/or intranasal insulin in MCI or mild AD.

### 4.2. Anxiety and Depression

Ng et al. found chronic ADPN reduction in mice associated with increased depressive and anxious behavior [45]. Furthermore, in rodents, the ADPN injection showed anxiolytic and antidepressant effects [45]. From a recent meta-analysis, patients with depression showed lower ADPN plasma levels compared to unaffected patients and an increase in levels after treatment of at least 12 months with antidepressants, suggesting time-dependent effects in antidepressant-induced alterations in ADPN levels [64]. It is probable that the overactivity of the hypothalamic-pituitary-adrenocortical axis contributes to depression through reduced serotonin availability in the brain. In fact, exogenous glucocorticoid drugs having side effects like depression appear to have an inhibitory effect on ADPN expression. However, on the contrary, patients with higher levels of ADPN also had higher levels of cortisol. In addition, ADPN levels follow a similar diurnal variation of cortisol, which suggests that they might be influenced by common regulatory factors. Furthermore, exogenous ADPN administration via intra-cerebroventricular leads to antidepressant-behavioral effects, and the antidepressant activity of the PPARγ agonists may be due to increased expression of ADPN [1].

### 4.3. Hypertension, Atherosclerosis, and Stroke

Previous studies demonstrated that hypertension causes severe damage to vascular endothelial cells, inflammation, and the loss of nitric oxide (NO) bioavailability, causing cerebral vascular dysfunction until subclinical brain infarcts and stroke [65].

The endothelium is essential to maintain the BBB [66] and to exert trophic effects on brain cells [67], while the deficit of NO derived from endothelial dysfunction can affect cerebral perfusion and lead to cognitive decline [68].

Many epidemiological studies showed that hypertension is correlated with the cognitive decline that mainly occurs in middle age and during the early stage of dementia [69]. Contrarily, some studies show that later-life hypertension may even help to prevent cognitive decline [70].

Consequently, hypertension may contribute to age-related cognitive dysfunction, and be linked to both VD [65,71] and to AD, increasing and accumulation Aβ amyloid. ADPN decreases the risk of hypertension and improves cognitive impairment by promoting NO release through AdipoR1 and AdipoR2 activation. Moreover, ADPN inhibits cerebral inflammatory response through AMPK/eNOS signaling pathway activation. In addition, ADPN could suppress amyloid-β in mice [41]. Consequentially, APN could decrease the risk of hypertension and improve vascular cognitive impairment.

Hypertension is a major, independent risk factor for atherosclerotic vascular disease.

Atherosclerosis is a progressive vessel disease that causes partial or total occlusion of large- or medium-sized arteries vessel lumen, and consequential cerebral hypoperfusion associated with an increased risk for ischemic stroke and dementia [72].

Chronic inflammation and an abnormal amount of lipids in the blood are involved in the development of atherosclerosis [73]. Most studies confirmed that ADPN may inhibit the release of pro-inflammatory cytokines and exert an effect on improving atherosclerosis in the brain by regulating atherogenic factors.

Stroke is a cerebral disease that causes vascular disorders, leading to decreased neurological function with an increased risk for VD and AD [74,75]. Immediately after a stroke, a sharp decline in cognition occurs (post-stroke dementia), else only after several years after stroke an accelerated cognitive decline appears [76]. Therefore, dementia represents a severe problem in stroke survivors. Various studies have evaluated the relationship between ADPN and stroke, but the results are contradictory. Some studies suggest that a low ADPN level is related to greater stroke risk [77,78], while other studies report that elevated ADPN levels may contribute to an increased risk of ischemic stroke [79]. In some other studies, there are no links between ADPN and stroke risk [80].

## 5. Adiponectin, Menopause and Cognitive Decline

Wennberg et al. [81] found that women had significantly higher levels of plasma ADPN, compared to men, as well as greater Aβ deposition. The association between higher plasma levels of ADPN and the female sex is interesting when related to BMI [81]. In women, high levels of ADPN are related to smaller hippocampal volume, and to poor performance in language and cognitive domains. In older women, high ADPN levels are related to a greater risk of MCI, due to an inverse correlation with estrogens.

The specific mechanism for gender differences in cognitive decline remains unclear. Irrespective of this, women have a greater risk of developing lifetime dementia [82]. Epidemiological studies suggest a relationship between midlife metabolism and cognitive performances [83,84]. During the menopause transition, many women may experience weight gain, associated with central fat deposition and consequently obesity [85,86].

Although more studies have shown that obesity is associated with impaired neurocognitive performance and structural changes in the brain, including brain atrophy and white matter disease [8], by contrast, there is some evidence that mild obesity protects against cognitive decline, especially in women, which is most likely explained by the highest endogenous estrogen levels [87,88]. Clinical evidence suggests that the reduction in estrogen that occurs both during the menopausal transition and after postmenopause can adversely affect brain function, particularly memory and verbal fluency [89].

Therefore, during the menopausal time that presents itself as a risk for dementia, in order to prevent and delay cognitive decline, it is required to identify both metabolic phenotype [90] and early biomarkers, such as peripheral indicators for cognitive decline risk. During the postmenopausal period, in both obese and insulin-resistant patients, serum ADPN levels are significantly reduced [90,91]. Indeed, it is known that in the postmenopausal period that low ADPN plasma levels are associated with an increased prevalence of metabolic syndrome, osteoporosis, and obesity [92]. No study has evaluated, during the menopausal period, the likely relationship about serum ADPN levels and cognitive performance.

In our recent study [93], we selected a population of menopausal women in order to evaluate the possible relationship between serum ADPN levels and cognitive performance. Considering that weight gain is often observed in menopause, we also evaluated the relationship between overweight or obesity, serum ADPN levels and cognitive performance. Hence, we have shown that there is a significant positive association between serum ADPN levels and better cognitive function in postmenopausal women. In particular, we demonstrated that a decline in estrogens during menopausal transition can adversely affect the brain, more specifically, in the aspects related to attention and executive functioning.

Although the changes in the attentional process and executive function are not heavy if compared to the changes in recent memory, they can, however, affect all cognitive performances [94,95]. In particular, attention is a function that derives from the activation of a complex neuronal network influencing the function of other brain networks, the result of which is allowing to perform more than one task at the same time [94,95]. Instead, executive function derives from integrative functions of higher cognitive processes which in turn influence both cognitive and behavioral components. This merge of functions, however, allows the management of the independent activities of daily life [87,88]. However, it is physiological that aging affects these mechanisms. Unfortunately, cognitive assessment is not always done during the menopause, and when it is performed, the cognitive evaluation is done using the Mini-Mental State Examination (MMSE) [96], which often is inadequate to identify MCI, probably due to the low sensitivity and the reduced number of items, as well as the absence of specific items that investigate executive functions. In addition, MMSE is known to be more specific for patients already suffering from dementia [96]. Thus, in our study, we used the Montreal Cognitive Assessment (MoCA test) test because we had the possibility of evaluating a greater number of cognitive domains [96]. In fact, the MoCA test is a more articulated but complete test, including the evaluation of complex executive, attention, visual, temporal-spatial functions, thus allowing the identification of even only minimal alterations of these functions [96]. Our findings found that serum ADPN level was the major determinant of attentional capacity, thus enabling us to speculate that the dosage of serum ADPN levels may represent an early serum marker of cognitive decline, thus emphasizing the importance of prevention. Our findings also suggest that serum ADPN levels influence cognitive performances independent of obesity. Consequently, lower levels of ADPN may be associated with cognitive dysfunction, also likely due to low-grade systemic inflammation that often is associated with MCI, as well as the obesity, that in turn, accompanied by a pro-inflammatory condition, stimulates the production of cytokines and thus generates a pathological spiral. In particular, our results highlight that more specialized and more punctual approaches are needed, especially for menopausal women, in whom cognitive evaluation is often not carried out, neither with psychometric tests nor with dosages of possible early markers of cognitive impairment, as the dosage for ADPN. Indeed, for menopausal women, the cognitive evaluation trough psychometric tests use represented a new diagnostic approach for the cognitive sphere, usually not evaluated. As also highlighted in some studies [97], offering new and long-term solutions reduces the risk of disability and expands knowledge by projecting research in new directions”.

In agreement with our study, Waragai et al. [23] showed that women are likely to have more cognitive impairment than men at the same level of neuropathology. They predict that this gender difference may be at least in part attributable to the decrease in BBB integrity in aging, in addition to other mechanisms, including hormonal differences.

Lastly, since ADPN is now a therapeutic possibility for many diseases [98], its use and/or the activation of its receptors could be a promising way of possible modulation also of cognitive decline.

## 6. Conclusions

**Figure 1:**
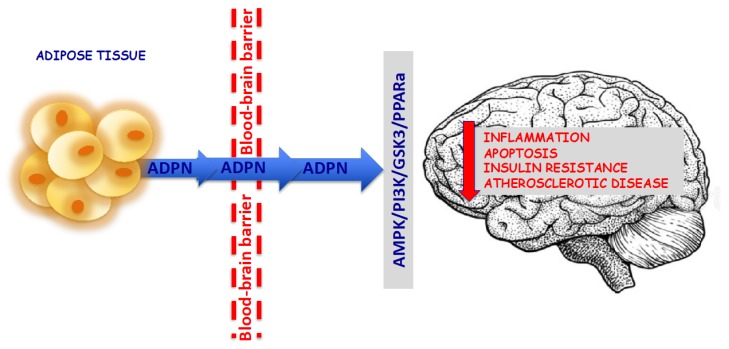
Adiponectin (ADPN) can be considered a molecular of interest in the search for new neuroprotective target for dementia.

Due to its strong protective effects on both the nervous system and peripheral tissues, ADPN can be considered a possible therapeutic medication to treat cognitive decline and metabolic syndromes. Therefore, many efforts have been made to identify classes of drugs that could function as ADPN receptor agonists. Unfortunately, to date, no one has discovered an ADPN receptor agonist, perhaps because the signal activation network is very complex and as such is difficult to imitate. Furthermore, ADPN cannot be orally administered due to its protein nature, while intravenous treatments of recombinant ADPN protein are costly. Alternative ways, such as lifestyle changes, activation of the ADPN pathway or ADPN receptor agonists or physical exercise, have been taken into consideration to increase peripheral ADPN levels and to transport ADPN across the BBB to exert beneficial effects on brain functions. Therefore, due to its likely protective roles, including insulin-sensitizing and its anti-inflammatory and anti-oxidative effects, ADPN could represent a possibility for preventing and treating dementia (Figure 1).
